# Blocking of tumor necrosis factor activity promotes natural repair of osteochondral defects in rabbit knee

**DOI:** 10.3109/17453670903350115

**Published:** 2009-10-01

**Authors:** Amu Kawaguchi, Hiroyuki Nakaya, Takahiro Okabe, Keiji Tensho, Masashi Nawata, Yoshitaka Eguchi, Yuuki Imai, Kunio Takaoka, Shigeyuki Wakitani

**Affiliations:** ^1^Department of Orthopaedic Surgery, Shinshu University School of MedicineMatsumotoJapan; ^2^Department of Orthopaedic Surgery, Sumitomo HospitalOsakaJapan; ^3^Department of Orthopaedic Surgery, Marunouchi HospitalMatsumotoJapan; ^4^Department of Orthopaedic Surgery, Osaka City University Graduate School of MedicineOsakaJapan

## Abstract

**Background and purpose** Osteochondral defects have a limited capacity for repair. We therefore investigated the effects of tumor necrosis factor (TNF) signal blockade by etanercept (human recombinant soluble TNF receptor) on the repair of osteochondral defects in rabbit knees.

**Material and methods** Osteochondral defects (5 mm in diameter) were created in the femoral patellar groove in rabbits. Soon after the procedure, a first subcutaneous injection of etanercept was performed. This single injection or, alternatively, 4 injections in total (twice a week for 2 weeks) were given. Each of these 2 groups was divided further into 3 subgroups: a low-dose group (0.05 μg/kg), an intermediate-dose group (0.4 μ g/kg), and a high-dose group (1.6 μ g /kg) with 19 rabbits in each. As a control, 19 rabbits were injected with water alone. The rabbits in each subgroup were killed 4 weeks (6 rabbits), 8 weeks (6 rabbits), or 24 weeks (7 rabbits) after surgery and repair was assessed histologically.

**Results** Histological examination revealed that the natural process of repair of the osteochondral defects was promoted by 4 subcutaneous injections of intermediate-dose etanercept and by 1 or 4 injections of high-dose etanercept at the various time points examined postoperatively (4, 8, and 24 weeks). Western blot showed that rabbit TNFα had a high affinity for etanercept.

**Interpretation** Blocking of TNF by etanercept enabled repair of osteochondral defects in rabbit knee. Anti-TNF therapy could be a strategy for the use of tissue engineering for bone and cartilage repair.

## Introduction

Osteochondral defects have a limited capacity for repair. Although the reason for this is still unclear, low progenitor cell numbers and/or low levels of growth factors are believed to play some part in this phenomenon. To promote repair, administration of various types of growth factors that function as anabolic factors—such as bone morphogenetic proteins (BMPs) (Sellers et al. 1997), insulin-like growth factors (IGFs) (Singh et al. 2007), fibroblast growth factors (FGFs) (Ishii et al. 2007), and combinations of them (Martin et al. 2001, Takahashi et al. 2005)—have been tested and found to be effective to some extent, though the effects of catabolic factors have not been well investigated.

Tumor necrosis factors (TNFs) and other pro-inflammatory cytokines are well-known catabolic factors in inflammation and tissue repair (Feldmann and Maini 1999, Goldberg et al. 2007). Anti-TNF therapy has been applied to various types of serious inflammatory disease, such as rheumatoid arthritis (Genovese et al. 2005), Crohn's disease (Camilleri 2007), ankylosing spondylitis (Maksymowych et al. 2005), and spondylarthropathy (Kruithof et al. 2005), as well as to degenerative disease of the intervertebral disc (Autio et al. 2006). Anti-TNF therapy is frequently used in patients with rheumatoid arthritis, and may promote regeneration of eroded joints (van der Heijde et al. 2006).

We evaluated the effects of subcutaneously injected etanercept (soluble TNF receptor; p75) at various doses and after various lengths of time on repair of 5-mm osteochondral defects made in the femoral patellar groove in rabbit knees.

## Material and methods

### Operative procedure

We used 133 mature Japanese white rabbits (Japan SLC Co. Ltd., Hamamatsu, Japan) weighing 2.9 (2.7–3.0) kg. The average age was 24 weeks. They were anesthetized by intramuscular injection of a mixture of ketamine (100 mg/mL, 0.6 mL/kg body weight) and xylazine (20 mg/mL, 0.3 mL/kg body weight). The patellar groove was exposed bilaterally through a parapatellar medial longitudinal incision and lateral subluxation of the patella. Osteochondral defects (5 mm diameter and 5 mm depth) were created, and then the patella was reduced and the wound closed in layers. Each rabbit underwent surgery in both knees simultaneously using the same procedure. Postoperatively, the animals were left uncasted and allowed free cage movement upon awakening. None of the rabbits limped after surgery.

### Etanercept administration

Administration of etanercept (Wyeth Pharmaceuticals, Madison, NJ, USA) was performed in 2 different ways, with either 1 or 4 injections. One group had only 1 subcutaneous injection whereas the other group had 4 such injections. The first injection was performed soon after the operation in each group. 4 injections were performed twice a week for 2 weeks. In addition, to evaluate the dose-dependency of any effects of etanercept, we established 3 subgroups with different doses of etanercept in 0.5 mL distilled water: 0.05 μg /kg (the low-dose group), 0.4 μg /kg (the intermediate-dose group), and 1.6 μg /kg (the high-dose group). Animals in the control group received only 1 injection of 0.5 mL water, and not 4 injections, since we did not expect the findings to differ markedly between them. The rabbits in each subgroup were killed 4, 8, or 24 weeks after surgery (6, 6, and 7 rabbits, respectively), and repair was assessed histologically using a scoring system.

### Histological assessment

The rabbits were killed by excessive intravenous injection of anesthetic agents at each time point. The distal femurs were cut sagittally in the center of the patellar groove and fixed in 10% formaldehyde, decalcified in 10% ethylenediaminetetraacetic acid, embedded in paraffin wax, cut into sections of 5 μm, and stained with hematoxylin and eosin or toluidine blue.

For evaluation of osteochondral defect repair, we designed a new histological grading score (Table 1) by modifying the scoring system previously reported by us (Wakitani et al. 1994). It involved 8 categories: (A) cell morphology, (B) matrix staining, (C) surface regularity, (D) thickness of the defect, (E) integration of repair tissue with the surrounding articular cartilage, (F) arrangement of repair cartilage, (G) remodeling of subchondral bone, and (H) effects on adjacent cartilage. The scores ranged from 25 points for normal tissue to 0 points for poorest repair. Sections were examined blind and scored independently by 3 of the authors, without knowing which group was being examined.

**Table:** Table 1. Histological scoring system for evaluation of repair of osteochondral defects. This was a modification of the scoring system reported by Wakitani et al. (2002)

		Score
1) Features of repair cartilage		
A) Cell morphology		
Hyaline cartilage		6
Mostly hyaline cartilage	> 3/4	5
Partly hyaline cartilage	1/4–3/4	4
Mostly fibro-cartilage	> 3/4	3
Partly fibro-cartilage	1/4–3/4	2
Mostly non-cartilage		1
None cartilage only		0
B) Matrix staining (metachromasia)		
Normal (compared to host)		4
Slightly reduced staining	> 3/4	3
Moderately reduced staining	1/4–3/4	2
Remarkably reduced staining	< 1/4	1
No metachromatic staining		0
C) Surface regularity (total smooth area compared to the whole area of the cartilage defect)		
Smooth	> 3/4	3
Moderate	1/2–3/4	2
Irregular	1/4–1/2	1
Severe irregular	< 1/4	0
D) Thickness of the defect (average thickness of reparative cartilage compared to that of surrounding cartilage)		
Normal	> 2/3	2
Moderate	1/3–2/3	1
Thin	< 1/3	0
E) Integration of repair tissue to the surrounding articular cartilage		
Both edges integrated		2
One edge integrated		1
Both edges not integrated		0
F) Arrangement of repair cartilage		
Column-like arrangement		2
Partly column-like arrangement		1
Disordered		0
2) Features of surrounding tissue		
G) Remodeling of subchondral bone		
Complete reconstruction		3
Continuous but incomplete reconstruction	2	
Discontinuous, greater than 50% reconstruction	1	
Discontinuous, less than 50% reconstruction	0	
H) Effect on adjacent cartilage (toluidine blue staining of adjacent cartilage adjacent to the edge of the defect)		
Normal (compared to host)		3
Slightly reduced staining	> 3/4	2
Remarkably reduced staining	1/4–3/4	1
Little or no metachromatic staining	< 1/4	0

### Western blot of rabbit TNF-α 

Anesthetized rabbits were administered 500 μg of lipopolysacharide (LPS; from *E. coli* serotype 026:B6; Sigma-Aldrich, St Louis, Mo, USA) in 500 μL PBS into the knee joints. 24 h after LPS administration, the rabbits were anesthetized and synovial fluid was collected by aspiration.

Collected synovial fluid was lysed in RIPA buffer containing 1 mM phenylmethylsulfonyl fluoride (PMSF) and protease inhibitor cocktail at 50 μL/mL (Sigma-Aldrich). Samples were centrifuged for 15 min at 15,000 rpm and the supernatants were transferred to new tubes. Then tubes containing the supernatant with or without 25 μg of etanercept were gently rotated for 60 min at 4ºC with 10 μL of a 50% slurry of Protein G Sepharose (Amersham Biosciences, Pittsburgh, PA, USA), which binds to the FC portion of etanercept and accelerates precipitation. After the treatment with or without etanercept, the tubes were centrifuged for 1 min at 2,000 rpm and the supernatants were transferred to new tubes. The precipitates and 20 μL of the supernatants were mixed with SDS polyacrylamide gel electrophoresis (PAGE) buffer and boiled for 10 min, then applied to each lane of a 4–20% SDS polyacrylamide gel (20 mA, low voltage for 90 min), and ultimately transferred to Immobilon-P PVDF membrane (Millipore). The membrane was blocked for 1 hour at room temperature with 5% skim milk (Difco) in Tris-buffered saline (TBS) followed by washing with TBS containing 0.1% Tween 20 (TBST) for 15 min; it was then incubated overnight with goat anti-rabbit TNF-α antibody (1:50 in blocking buffer; Fitzgerald Industries International, Concord, MA, USA) at 4ºC. After washing twice with TBST for 15 min, the membrane was incubated with peroxidase-conjugated rabbit anti-goat antibody (1:500; Dako, Glostrup, Denmark) for 30 min. After washing twice again with TBST for 15 min, the membrane was developed with ECL Plus reagent (Amersham Biosciences).

### Statistics

Differences in histological score between the control group and each etanercept treatment group at various time points postoperatively (4, 8, and 24 weeks) were tested by the post hoc Scheffe test using StatView-J version 5.0 (SAS Institute, Cary, NC).

## Results

### Histological observations

**Figure 1.**
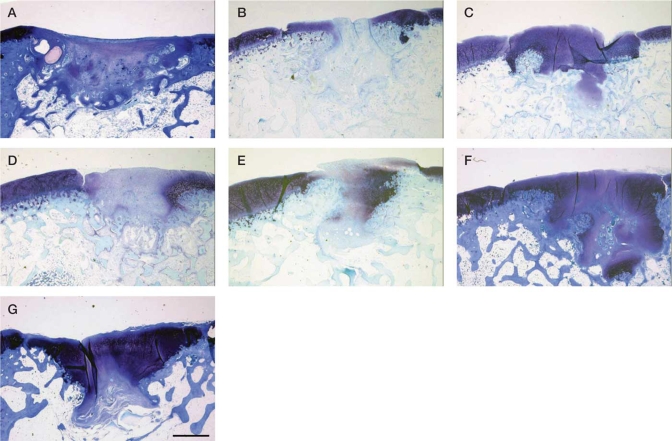
Representative histological images of osteochondral defects after 4 weeks, stained with toluidine blue (magnification ×20). A: control group; B: 1 injection, low dose; C: 1 injection, intermediate dose; D: 1 injection, high dose; E: 4 injections, low dose; F: 4 injections, intermediate dose; G: 4 injections, high dose. Large areas of metachromatic staining were observed in C, D, E, F, and G.

4 weeks after surgery the defects were found to be covered with fibrous tissue, with partial and weak metachromasia in the control group. In all the etanercept groups, especially in the intermediate and high-dose subgroups that received either 1 or 4 injections, metachromasia was wider and stronger than in the control group. Repair of subchondral bones was better in all etanercept groups than in the control group (Figure 1). These findings suggest that etanercept promoted early subchondral bone remodeling of osteochondral defects.

8 weeks after surgery, the defects were covered with fibrous tissue with partial metachromasia, and they were thinner than those at 4 weeks. Subchondral bone repair had been promoted in all groups, including the control group. Repair was good with 1 injection in the high-dose subgroup, though in the other groups there were no discernible differences in repair from that in the control group (data not shown).

**Figure 2.**
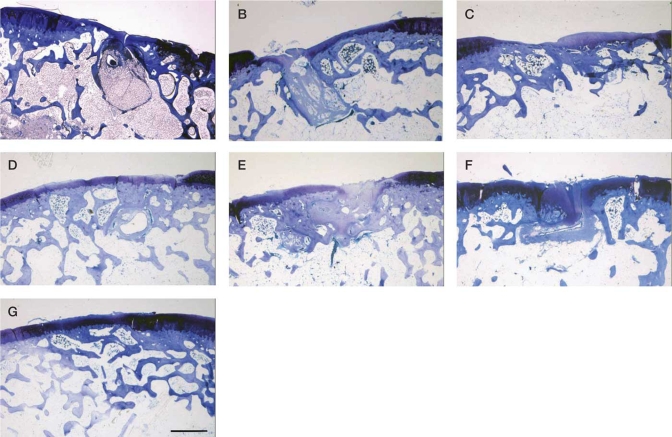
Representative histological images of osteochondral defects after 24 weeks, stained with toluidine blue (magnification ×20). A: control group; B: 1 injection, low dose; C: 1 injection, intermediate dose; D: 1 injection, high dose; E: 4 injections, low dose; F: 4 injections, intermediate dose; G: 4 injections, high dose. Nearly complete subchondral bone remodeling and continuous cartilage regeneration were found in F and G, while subchondral bone remodeling remained discontinuous in A, B, and C.

24 weeks after surgery, repair of subchondral bone was almost complete, and the repaired cartilage was slightly thinner than the adjacent normal cartilage (Figure 2). The cell morphology and matrix staining indicated good repair with 4 injections in the intermediate and high-dose subgroups.

On histological evaluation of some samples in the intermediate and high-dose subgroups, promotion of repair was so dramatic that the repair tissue looked almost like complete hyaline cartilage histologically.

Adjacent cartilage did not show any degeneration in any of the samples examined, indicating that etanercept does not affect metabolism of normal cartilage.

### Histological scoring system

The differences in histological scores in the control group and in the 6 different subgroups at various time points postoperatively (4, 8, and 24 weeks) showed statistically significantly better repair with 4 injections in the intermediate-dose subgroup and 1 and 4 injections in the high-dose subgroups than in the other groups (Table 2).

**Table:** Table 2. Histological scores by group. Figures are mean (SD)

Dose	No. of injections	Overall difference	4 weeks (6 rabbits)	8 weeks (6 rabbits)	24 weeks (7 rabbits)
Control	1		14 (1.1)	14 (3.0)	14 (3.5)
Low	1	none	15 (2.8)	15 (2.4)	16 (3.8)
	4	none	13 (3.4)	13 (3.9)	13 (3.3)
Intermediate	1	none	17 (1.8)	16 (4.2)	14 (3.4)
	4	yes	16 (2.0)	17 (5.2)	19 (2.3)
High	1	yes	17 (2.6)	19 (3.7)	16 (2.6)
	4	yes	16 (2.7)	17 (1.1)	18 (3.6)

### Western blot

**Figure 3.**
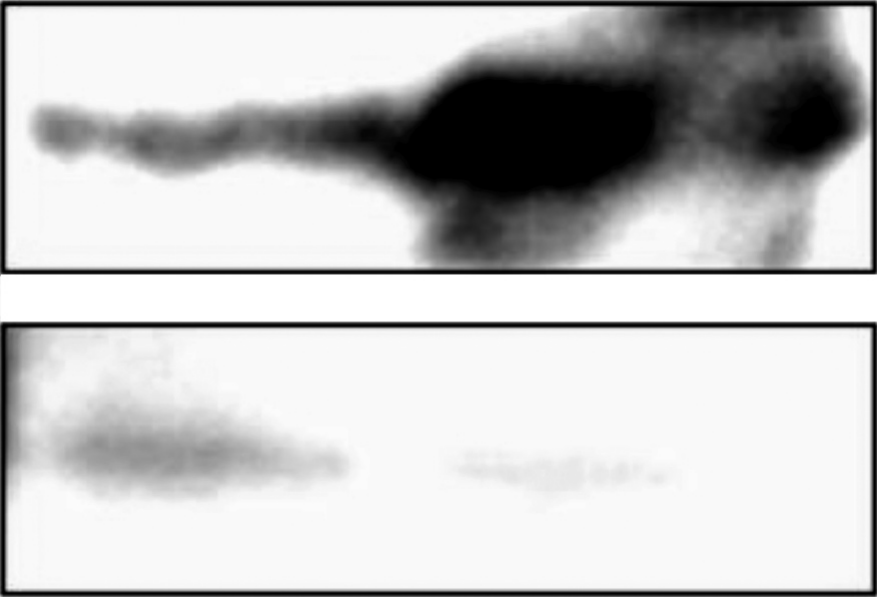
Western blot of TNF-α Samples were prepared from LPS-induced synovial fluids with or without eternacept (right-hand and left-hand lanes, respectively). Precipitates (upper panel) and supernatants (lower panel) of each sample were separated by SDS-PAGE and the presence of TNF-α detected by sandwich technique using polyclonal antibodies.

Western blot analysis indicated that the synovial fluid from joints with LPS-induced arthritis contained TNF-α (Figure 3). When we treated the samples with etanercept, the TNF-α present in precipitates was dramatically increased while that in supernatants was reduced at the same time. These findings confirmed that etanercept has a substantial affinity for rabbit intra-articular TNF-α.

## Discussion

The natural process of repair of 5-mm osteochondral defects in the patellar groove of rabbit knees was promoted by 4 subcutaneous injections of intermediate-dose etanercept and by 1 or 4 injections of high-dose etanercept at the various time points examined postoperatively. The repair process was promoted even with 1 injection just after surgery, indicating that blocking of TNFs early on in the repair process is important. However, histological examination at 24 weeks showed that 4 injections were more effective than 1 injection. These findings suggest that TNFs are involved in the process of cartilage and subchondral bone repair at least 2 weeks after creation of defects, and that blockade of TNFs in this period leads to better repair of defects. We assumed that TNFs exhibit effects early after the creation of defects, and we therefore decided to block TNF activity in the first 2 weeks. Because we hypothesized that TNFs might show effects early within the 2-week postoperative period, we included 1-injection groups. We did not include groups that received etanercept injection for more than 2 weeks. Further investigation may be required to determine the appropriate period of etanercept administration.

This study revealed dose-dependent effects of etanercept on the process of repair of osteochondral defects in the rabbit knee. The intermediate dose of etanercept was 40 μg/kg, which approximately matches the clinically used dose in humans (2.5 mg) assuming an average human body weight of 60 kg. On the other hand, administration of 160 μg/kg in the high-dose subgroups, equivalent to 9.6 mg in humans and thus corresponding to administration of an overdose, showed effects on cartilage repair similar to those seen in the intermediate-dose subgroups, while the low dose (5 μ/kg)—equivalent to 0.3 mg in humans—had only limited effects.

When tissues are injured, catabolic cytokines including TNFs are released, evoke inflammation, and function in the removal of destroyed tissue. However, it is likely that strong inflammation interferes with tissue repair. Previous studies have revealed enhancement of repair by inhibition of the effects of TNFs on bone formation in vivo and in vitro (Hashimoto et al. 1989, Zhou et al. 2006) and on meniscus in vitro (McNulty et al. 2007). As found in our study, an antagonist of TNFs promoted repair of osteochondral defects. TNFs may interfere with the mobilization, proliferation, and/or differentiation of mesenchymal osteochondro-progenitor cells, though their contribution to cartilage and bone repair is controversial (Fukui et al. 2006)

Our scoring system was a modified version of a system previously reported for the evaluation of rabbit osteochondral defect repair. The principal modifications we made concerned remodeling of subchondral bone and degenerative change in adjacent normal cartilage, which are important factors in histological evaluation of cartilage repair. Complete remodeling of subchondral bone is necessary for cartilage repair, both mechanically and biologically. It is important to determine whether operative procedures and drugs affect not only repair of tissue but also adjacent normal cartilage. It is important to emphasize that good reconstruction of subchondral bone was obtained at 4 weeks with both 1 and 4 injections in the intermediate and high-dose subgroups. It appears that blocking of TNFs may be more effective in improving bone repair than cartilage repair at 4 weeks. The International Cartilage Repair Society (ICRS) cartilage repair assessment score (O'Driscoll et al. 1988) was established for clinical use, but is not good for small animal experiments. This system is for assessment by arthroscopic appearance and cannot be used for assessment of subchondral bone and matrix staining of the adjacent normal cartilage.

Our findings suggest that cytokine therapy may be useful for promoting cartilage repair. Bone marrow-stimulating operative procedures such as abrasion, drilling, and microfracture (Kreuz et al. 2006) and tissue engineering techniques are sometimes performed in patients with osteoarthritis or osteochondritis dissecans (Wakitani et al. 2002), and blocking of TNF activity may promote cartilage repair in conjunction with these techniques. The dosage of TNF-blocking agents is of importance, since our findings indicate that incorrect dosages may result in insufficient cartilage repair or in adverse effects.

Small defects less than 3 mm wide have been reported to be spontaneously repaired in rabbits (Lietman et al. 2002). We made osteochondral defects 5 mm in diameter and 5 mm deep in large, fully mature rabbits since it appeared that this might enhance differences in therapeutic effects between the conditions studied. The control group did not exhibit repair.

There are 2 receptors for TNFs, p55 and p75. Etanercept is the soluble TNF receptor, p75, which binds to both TNF-α and TNF-β in humans. There have been no previous reports that etanercept binds to rabbit TNFs. Here we found that etanercept binds to rabbit TNF-α.

It may be possible to use etanercept, systemically or locally, in the repair of osteochondral defects in humans. However, because our study was a preliminary one using a rabbit model, further experiments will be necessary to confirm the usefulness of etanercept in promoting the repair of osteochondral defects in humans.
